# The impact of duration below target mean arterial pressure value on mortality in critically ill patients

**DOI:** 10.12669/pjms.41.10.12574

**Published:** 2025-10

**Authors:** Mehmet Cihat Demir, Erdinc Senguldur, Kudret Selki

**Affiliations:** 1 Mehmet Cihat Demir, Department of Emergency Medicine, School of Medicine, Duzce University, Duzce, Turkiye; 2 Erdinc Senguldur, Department of Emergency Medicine, School of Medicine, Duzce University, Duzce, Turkiye; 3 Kudret Selki, Department of Emergency Medicine, School of Medicine, Duzce University, Duzce, Turkiye

**Keywords:** Critically ill patients, Emergency department, Mean arterial pressure, Mortality

## Abstract

**Objective:**

Literature lacks exploration of how the duration of low mean arterial pressure (MAP) affects mortality. This study aimed to determine whether the duration of MAP below 65 mmHg is associated with mortality in critically ill patients (CIPs).

**Methodology:**

A prospective observational study was conducted with patients who were admitted to the emergency department (ED) from July 1 to December 31, 2023, with a follow-up in the ED critical care unit for at least six hours. The average MAP and the total duration that the MAP remained below 65 mmHg (ΔtMAP<65) during this period were calculated.

**Results:**

In the study with 103 CIPs, ΔtMAP<65 was significantly higher in patients who died within 48 hours (247 [210-300] vs. 90 [30-135]) (p<0.001). ΔtMAP<65 was also significantly higher in patients who died within 30 days (195 [150-270] vs. 45 [15-75]) (p<0.001). Average MAP and ΔtMAP<65 highly predict 48 hours mortality, with AUC values of 0.887 and 0.957, respectively. ΔtMAP<65 for ≥180 minutes increased the risk of 48 hours mortality 173.25 (36.58-820.40) times. Average MAP and ΔtMAP<65 predicted 30 days mortality with AUC values of 0.890 and 0.963, respectively. ΔtMAP<65 for ≥135 minutes increased 30 days mortality 166.66 (20.78-1336.66) times.

**Conclusions:**

This study shows that the duration of MAP below 65 mmHg is associated with mortality in CIPs. When ΔtMAP<65, lasting over 135 minutes significantly increases the 30 days mortality risk. If it exceeds three hours, the 48 hours mortality risk also rises substantially. Monitoring cumulative hypotension duration in critical care may enhance survival through earlier interventions.

## INTRODUCTION

Critically ill patients (CIPs) are one of the challenging groups in emergency department (ED) practice. Keeping arterial blood pressure (BP) under control is one of the most crucial steps in CIPs resuscitation. Hypotension is a frequently encountered condition in CIPs. Due to low arterial pressure, tissue and organ perfusion may be impaired and organ damage and failure may develop.^1^ Mean arterial pressure (MAP) is a parameter determined by cardiac output and peripheral arterial resistance and is frequently used in critical patient management to evaluate tissue and organ perfusion.^2^,^3^ In managing patients with sepsis, which constitutes a large proportion of CIPs, it aims to keep MAP above 65 mmHg.^4^-^7^

In conditions such as heart failure, chronic liver disease, and pulmonary thromboembolism, studies have shown that low MAP values are associated with increased mortality.^2^,^3^,^8^-^10^ However, unlike sepsis, no specific lower limit has been identified. Fluid therapy and vasopressors/inotropes are used to increase MAP. Intensive fluid treatments may cause volume overload. High doses of inotropic medications are proarrhythmic. Although it is critical to increase MAP in CIPs, aggressive treatments may lead to poor outcomes.^10^-^12^ Therefore, The Surviving Sepsis Campaign Guidelines strongly recommend an initial MAP of 65 mmHg in septic shock patients rather than higher MAP values.^7^ On the other hand failure to raise MAP rapidly enough may prolong the time tissues and organs are exposed to hypoxia and worsen the prognosis.

There is a significant knowledge gap in the literature regarding studies examining the relationship between the duration of low MAP and mortality. This study aimed to determine whether the duration of MAP remaining below 65 mmHg is associated with mortality in CIPs. This will provide new evidence regarding resuscitation strategies in CIPs and contribute to a better understanding of the importance of treatment protocols in these patients.

## METHODOLOGY

This is a prospective, single-center, observational study. It was conducted in the ED of a tertiary university hospital in Turkiye, which had approximately 100,000 annual admissions.

### Ethical Approval:

The study was initiated after local ethics committee approval (Approval ID: 2023/95, dated June 19, 2023) was obtained.

The study was conducted on patients who admitted to the ED during the six months between July 01, 2023 and December 31, 2023 and were decided to be treated in the emergency intensive care unit by the physician after triage assessment. In the ED where the study was conducted, a triage system coded with three colors is applied. The red triage color is used for CIPs, and these patients are followed up in the ED critical care unit. The term ‘critically ill’ refers to patients with impaired vital signs, a high risk of death if left untreated, and a potential for recovery.^13^

BP is measured every 15 minutes from the brachial artery using an automatic machine (Bionet model: BM07) with the help of an upper arm cuff in patients followed up in the ED critical care unit. The MAP value of the patients is routinely calculated by the formula diastolic BP + (systolic - diastolic BP)/3.^8^ In the ED where the study was performed, the necessary fluid and inotropic/vasopressor medication support are given to hypotensive patients by the physicians who follow the patients by the guidelines of the diseases causing hypotension.^7^,^14^ This study was conducted entirely observationally. No intervention was made in the diagnosis and treatment processes of the patients. Age and gender information, vital signs at admission, comorbid diseases and the diagnosis made in the ED were noted on the study forms.

### Selection of participants and study protocol:

Patients included in the study were those who were 18 years of age or older, admitted to the ED with a red triage color, and provided informed consent either personally or through a first-degree relative. Additionally, patients were required to have been monitored in the ED for at least six hours and experienced a MAP of less than 65 mmHg at least once during this period. When MAP was first detected below 65 mmHg, an immediate repeat measurement was performed to confirm the finding.

### Exclusion Criteria:

**Fig.1:**
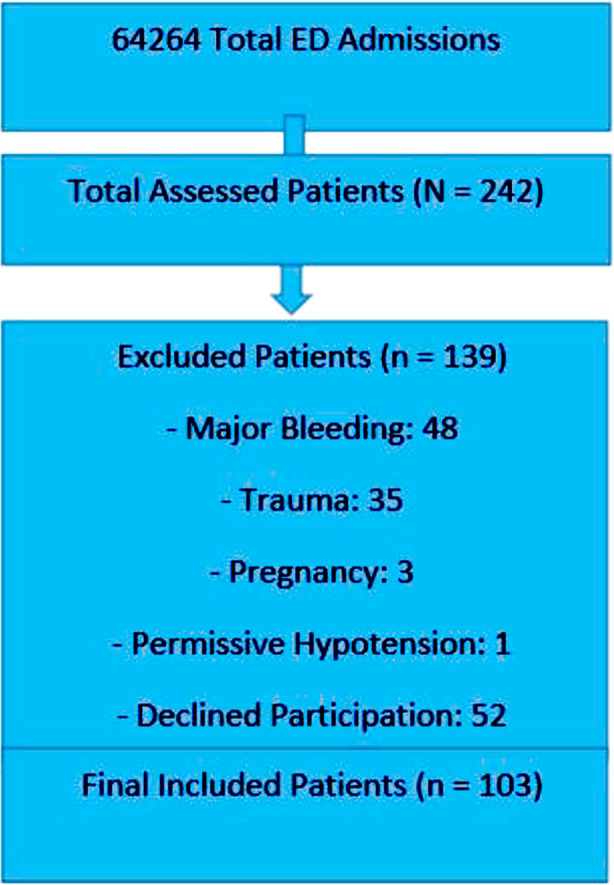
Selection and Exclusion of Study Participants.remained below 65 mmHg.

Patients were excluded if their hypotension was due to significant bleeding, such as gastrointestinal tract bleeding, intra-abdominal bleeding, or hemothorax. Trauma patients and pregnant individuals were also excluded. Furthermore, patients whose primary physician opted for a permissive hypotension strategy were not included in the study. Included and excluded patients are shown in Fig.1.

BP was measured every 15 minutes for the patients included in the study. MAP was calculated for each BP value measured. All patients’ BP and MAP values were recorded on the survey forms, as well as the time of the measurements. The six hours average MAP value (average MAP) and the total minutes during the six-hours follow-up that MAP remained below 65 mmHg (ΔtMAP<65) were calculated and recorded on the study forms. The ΔtMAP<65 calculation was obtained by summing the 15-minute periods between the measurements in which MAP<65 was detected. Consecutive hypotensive readings (MAP <65 mmHg) were considered part of the same ongoing episode, and the total duration was calculated cumulatively without resetting for each new reading.

We obtained and recorded 48 hours and 30 days mortality data for these patients using the hospital’s computer system. For those patients whose mortality information could not be accessed through the system, we contacted their relatives using the registered telephone numbers to gather the necessary mortality results.

Descriptive statistical data of the patients included in the study regarding demographic data, vital signs, diagnosis of sepsis, MAP at admission, average MAP, and ΔtMAP<65 were generated. Mortality groups were defined according to 48 hours mortality and 30 days mortality results. Comparative statistical data were generated between mortality groups regarding demographic data, vital signs, MAP at admission, average MAP, and ΔtMAP<65.

### Statistical analysis:

Numerical variables were summarized by median (interquartile range [IQR], 25th and 75th percentile) and categorical data by frequency and percentage. Conformity to normal distribution was analyzed using the Shapiro-Wilk test, Kolmogorov-Smirnov test, and histogram. Continuous data were compared between two groups using the Mann-Whitney U test. Pearson’s Chi-square test or Fisher’s Exact test was used to compare two categorical variables. Statistical software SPSS version 23 (SPSS Inc., Armonk, NY) was used for these analyses. The receiver operating characteristics (ROC) curve was analyzed using the Rstudio version (pROC package). Sensitivity, specificity, area under the curve (AUC), positive predictive value (PPV), and negative predictive value (NPV) were calculated to assess the predictive performance of average MAP and ΔtMAP in detecting 48-hour mortality and 30-day mortality. Optimum cut-off values were determined by the Youden index J point, and risk analysis was performed. The odds ratio (OR) was presented with a 95% confidence interval (CI). The significance level was accepted as p < 0.05.

### Sample size and power analysis:

A post-hoc power analysis was conducted to determine whether the sample size was sufficient to detect a significant difference in MAP between patients who survived and those who died within 30 days. The effect size (Cohen’s d) was calculated based on the observed means and standard deviations. Using an independent sample t-test model, the statistical power was found to be 1.0 (100%), indicating that the sample size was more than adequate. Sensitivity analysis was also performed by increasing the standard deviations by 50%, which resulted in a reduction of the effect size to 0.97, with a corresponding increase in the minimum required sample size to 22 patients for 80% power. Despite this increased variability, our study’s sample size (N = 103) remained sufficiently powered.

## RESULTS

There were 64264 ED admissions during the study period. Of these, 103 patients met the criteria and were included in the study. The median age of the patients was 73 years, and 48.5% (n=50) were female. The median value of average MAP was 67.20 (61.80-72.20) mmHg, and ΔtMAP<65 was 120 (60-225) minutes. Descriptive statistical data of the patients included in the study in terms of demographic data, vital signs, sepsis diagnosis, MAP at admission, average MAP, and ΔtMAP<65 are shown in Table-I.

**Table-I:** Study Characteristics (n=103).

Parameter	Median (IQR 25-75)
Age, years	73 (66-81)
Gender, female, n (%)	50 (48.5)
Systolic arterial blood pressure (mmHg)	93 (76-113)
Diastolic arterial blood pressure (mmHg)	61 (46-73)
Pulse (bpm)	96 (86-116)
Sepsis (yes), n (%)	74 (71.8)
MAP at admission (mmHg)	72.33 (57.33-84.66)
Average MAP in 6 hours (mmHg)	67.20 (61.80-72.00)
ΔtMAP<65 (minutes)	120 (60-225)

MAP: mean arteriel pressure; ΔtMAP<65: Total minutes during the six-hours follow-up that MAP remained below 65 mmHg; IQR: interquartile range (25th-75th percentile).

In the study, the average MAP value of patients who died within 48 hours after ED admission was significantly lower than those who survived within 48 hours (60.00 [56.62-63.75] vs 70.50 [66.00-75.00]) (p<0.001). The ΔtMAP<65 value was significantly greater in the group of patients who died within 48 hours (247 [210-300] vs 90 [30-135]) (p<0.001). When grouped in terms of 30 days mortality, average MAP was significantly lower in the mortality group (63.00 [58.50-67.35] vs. 72.20 [69.50-77.25]), and ΔtMAP<65 was significantly greater (195 [150-270] vs. 45 [15-75]) (p<0.001). Comparative statistics of 48 hours and 30 days mortality groups in terms of demographic data, vital signs, MAP at admission, average MAP, and ΔtMAP<65 are shown in Table-II.

**Table-II:** Evaluation of study characteristics in terms of 48-hour mortality and 30-day mortality.

Parameter	48h mortality-Survived (n=67)	48 h mortality- Deceased (n=36)	p	30d mortality- Survived (n=41)	30d mortality Deceased (n=62)	p
Age (year)	73 (66-79)	73(65-84)	0.573	71 (62-79)	74 (67-83)	0.125
Gender (female)	35 (52.2)	15 (41.7)	0.262	22 (53.7)	28 (45.2)	0.533
Systolic arterial blood pressure (mmHg)	94 (83-123)	86 (72-108)	0.157	99 (86-124)	88 (72-108)	0.037
Diastolic arterial blood pressure (mmHg)	61 (45-76)	60 (47-69)	0.291	61 (51-76)	59 (43-71)	0.122
Pulse (bpm)	94 (81-109)	105 (90-124)	0.023	93 (76-104)	100 (90-120)	0.020
MAP at admission (mmHg)	72.33 (60.00-85.66)	72.33 (53.58-83.91)	0.222	75.00 (63.50-92.50)	70.00 (54.08-84.00)	0.066
Average MAP in 6 hours (mmHg)	70.50 (66.00-75.00)	60.00 (56.62-63.75)	<0.001	72.20 (69.50-77.25)	63.00 (58.50-67.35)	<0.001
Δt MAP<65 (minutes)	90 (30-135)	245 (210-300)	<0.001	45 (15-75)	195 (150-270)	<0.001

MAP: mean arteriel pressure; ΔtMAP<65: Total minutes during the six-hours follow-up that MAP remained below 65 mmHg; Variables are shown as n (%) or median (IQR, 25th-75th). p < 0.05 was considered significant.

**Fig.2:**
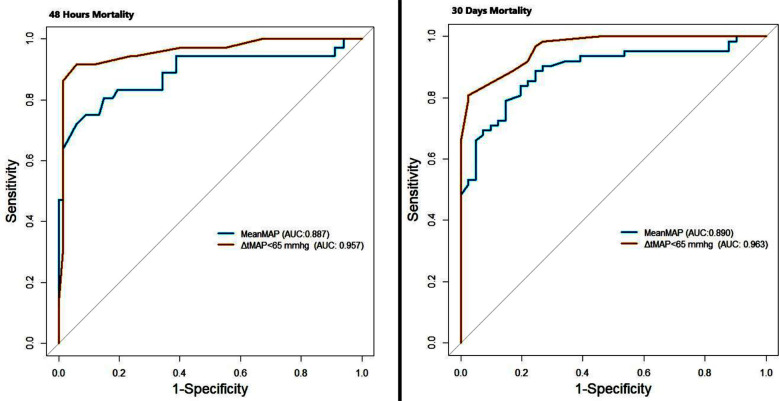
Receiver operating characteristic (ROC) curves of the six-hours average MAP value (MeanMAP) and ΔtMAP<65 values for 48 hours and 30 days mortality. *MAP: mean arteriel pressure; ΔtMAP<65: Total minutes during the six-hours follow-up that MAP remained below 65 mmHg*.

The performance, sensitivity, specificity, positive and negative predictive values, cut-off values, and odds ratio values of Average MAP and ΔtMAP<65 in predicting 48 hours and 30 days mortality are shown in Table-III. ROC curves prepared for 48 hours and 30 days mortality with average MAP and ΔtMAP<65 values are given in Fig.2. In the study, the predictive power of average MAP and ΔtMAP<65 for 48 hours mortality was relatively high, with AUC values of 0.887 and 0.957, respectively. Average MAP ≤63 mmHg increased the 48 hours mortality risk by 30.5 (9.873-94.226). ΔtMAP<65 with a duration of ≥180 minutes increased the 48 hours mortality risk 173.25 (36.58-820.40) fold. Average MAP and ΔtMAP<65 predicted 30 days mortality. AUC values were 0.890 and 0.963, respectively.

**Table-III:** Performance, odds ratios, sensitivity, specificity and predictive values of average MAP and ΔtMAP<65 values.

	AUC (%95 CI)	OR (%95 CI)	Sensitivity	Specificity	PPV	NPV	p
***Mortality in 48 hours: Yes***							
Average MAP	0.887 (0.809-0.966)	30.50 (9.87-94.22) (For ≤ 63 mmhg)	94.03%	72.2%	86.3%	86.70%	**<0.001**
ΔtMAP (minutes)	0.957 (0.913-1.000)	173.25 (36.58-820.40) (For ≥180 minutes)	91.67%	94.03%	89.19%	95.45%	**<0.001**
***Mortality in 30 days: Yes***							
Average MAP	0.890 (0.827-0.953)	17.18 (6.34-46.57) (For ≤ 68 mmhg)	85.37%	79.03%	72.92%	89.09%	**<0.001**
ΔtMAP (minutes)	0.963 (0.933-0.992)	166.66 (20.78-1336.66) (For ≥ 135 minutes)	80.65%	97.56%	98.04%	76.92%	**<0.001**

AUC = area under the curve; OR = odds ratio; CI = confidence interval; PPV = positive predictive value; NPV = negative predictive value. MAP: mean arterial pressure; ΔtMAP<65: Total minutes during the six-hours follow-up that MAP remained below 65 mmHg. Bold values indicates p < 0.05 was considered significant.

## DISCUSSION

In our study, both lower six-hour average MAP values and longer cumulative duration of hypotension (ΔtMAP<65) were strongly associated with increased 48-hour and 30-day mortality. Specifically, patients with ΔtMAP<65 exceeding 135 minutes had a 166-fold higher risk of 30-day mortality, and those with ΔtMAP<65 exceeding 180 minutes had a 173-fold higher risk of 48-hour mortality. These findings indicate that the cumulative burden of hypotension, rather than admission MAP alone, is a key determinant of outcomes CIPs.

Our results are in line with prior evidence emphasizing the prognostic value of maintaining adequate MAP. Maheshwari et al. showed that ICU hypotension was associated with increased in-hospital mortality in septic patients.^1^ Similarly, Lee et al. reported that MAP thresholds below 65 mmHg predicted worse 28-day survival in sepsis.^15^ More recently, Zuin et al. demonstrated that mean arterial pressure predicted short-term deterioration in patients with acute pulmonary embolism.^8^ These studies highlight the prognostic importance of MAP, yet most focused on baseline values or ICU populations. By contrast, our study specifically evaluated the cumulative duration of hypotension during early ED resuscitation, thereby providing novel evidence for emergency department protocols. In line with regional data, Senguldur et al. also demonstrated that dynamic hemodynamic parameters such as lactate clearance were predictive of short-term outcomes after CPR, further supporting the integration of time-dependent measures into early critical care management.^16^

MAP control is one of the key points in CIPs management. Avoiding hypotension and increasing MAP above target values are central to CIP resuscitation and cause-directed therapies.^17^ Prior research has tried establishing target MAP goals.^3^,^8^-^11^,^15^,^18^ The threshold MAP value recommended in the guideline for sepsis patients is 65 mmHg.^7^ The guideline includes a target MAP value of 65 mmHg for patients who achieved return of spontaneous circulation (ROSC) after cardiopulmonary resuscitation (CPR) following cardiac arrest.^19^ It has been investigated whether higher threshold values positively affect survival in various diseases, and most studies have not achieved lower mortality results with MAP targets above 65 mmHg.^9^-^11^ The target MAP value threshold in CIPs is still controversial. In this study, the median value of the six-hours average MAP of critically ill patients followed up in the ED was 67.20 (61.80-72.00) and was compatible with the literature targets.

Numerous studies have indicated that a low MAP is linked to a higher mortality rate in CIPs. Some research focuses on the correlation between average MAP values during follow-up and mortality rates,^12^,^15^ while other studies examine the impact of MAP measured at the time of admission on patient mortality.^2^,^3^,^8^ In this study, patients who died in the 48-hour and 30-day mortality groups had a significantly lower median value for the six-hours average MAP. Average MAP values below the cut-off values identified by ROC analysis (63 and 68 mmHg, respectively) significantly increased the mortality risk for the 48-hour and 30-day period by 30 and 17 times, respectively. MAP calculated at admission did not differ substantially between the 48-hour and 30-day mortality groups. The fact that the MAP value at the time of admission did not make a difference in terms of mortality reveals the importance of interventions performed in the ED. The MAP value at the time of admission is essential in the initiation of treatment and in determining the treatment strategy. Average MAP, which increases in parallel with the quality of the treatment, and ΔtMAP<65, which decreases inversely, are determinants of the patient’s prognosis.

Patients with sepsis constitute a large portion of CIPs.^2^,^12^,^20^ Sepsis and septic shock are clinical conditions with high mortality risk.^21^,^22^ In our study, 71.8% of the patients were followed up due to sepsis. In the initial resuscitation section of the Surviving Sepsis 2021 guideline, it is emphasized that sepsis and septic shock are medical emergencies, and treatment should be initiated immediately. Another recommendation of the guideline is to hospitalize patients with sepsis and septic shock in intensive care within the first six hours.^7^ The goal of initiating treatment without delay and reaching the targeted MAP values as soon as possible is also valid for non-sepsis CIPs.^23^-^25^ Our study observed that MAP remained below 65 mmHg in 120 minutes of six-hours ED follow-up of CIPs. This shows how compelling blood pressure regulation, especially the fight against hypotension, can be despite appropriate treatment.

The fact that ED physicians continuously encounter new patients while following CIPs makes the follow-up of CIPs in the ED extremely challenging. Especially in countries like Türkiye, where ED admissions are high and ED crowding is common, long-term follow-up of CIPs in ED conditions will decrease the quality of treatment. We think that the primary determinant of patient outcomes is not the delay in ICU admission itself but rather the timely initiation of appropriate supportive treatments. However, ED’s are not ideal environments for the prolonged management of critically ill patients. The high patient turnover and excessive workload in the ED can lead to suboptimal treatment implementation despite adherence to protocols.

While initial supportive measures can begin in the ED’s critical care unit, the limitations of this setting-including resource constraints, overcrowding, and concurrent management of multiple critically ill patients-can negatively impact the effectiveness and continuity of care. Transferring CIPs to ICUs as early as possible will facilitate ED patient management and contribute positively to patient surveys.^7^ While previous studies have emphasized maintaining MAP above threshold values in CIPs, there is a lack of research on how the duration of low MAP affects mortality. Our study demonstrates that longer hypotension increases mortality risk. The findings of our study showed that the 30-day mortality risk increased 166-fold when ΔtMAP<65 exceeded 135 minutes, and the 48-hour mortality risk increased 173-fold when ΔtMAP<65 exceeded three hours. We can say that MAP should be increased above 65 mmHg in the first two hours in order to prevent the ΔtMAP<65 duration of CIPs to exceed 135 minutes and to prevent it from dropping again. In patients whose MAP cannot be increased above 65 mmHg within the first three hours, the 48-hour mortality risk significantly escalates.

Potential confounding factors, including underlying comorbidities, variations in resuscitation strategies, and differences in treatment protocols during the ED stay, may have influenced the observed associations. Although these factors appeared balanced across mortality groups, they should still be acknowledged as possible contributors to patient outcomes.

This study provides new evidence that the cumulative duration of hypotension (ΔtMAP<65) is a strong predictor of short-term mortality in CIPs managed in the ED. Unlike most previous research that focused on baseline MAP values or ICU populations, our findings emphasize the importance of time-dependent monitoring during early resuscitation in the ED. Clinically, this highlights the potential for incorporating cumulative hypotension monitoring into ED protocols to guide timely interventions and improve outcomes.

### Strength of the study:

The major strength of our study is its prospective design with standardized monitoring and robust outcome measures. However, further multi-center studies with larger cohorts and longer follow-up are needed to validate these thresholds, explore potential interventions to reduce ΔtMAP<65, and assess their impact on long-term outcomes.

### Limitations:

The first limitation of our study is that it is a single-center study. The second limitation is that non-invasive BP measurement was performed, not invasive continuous arterial blood pressure measurement, which is the gold standard. The same method was applied to all patients in order not to disrupt the flow of the study. The third limitation is that the study did not consider the circadian rhythm of arterial blood pressure. The fourth limitation is the excess of patients who did not consent to the study. The fifth limitation is that although the study included a six hours follow-up, the ED follow-up of the patients lasted longer due to insufficient intensive care beds in our hospital, which may have caused a bias in mortality.

The sixth limitation is that a pre-determined sample size calculation was not performed before data collection. Instead, the study was conducted over a fixed six-month period, and all eligible patients meeting the inclusion criteria were enrolled. Although a post-hoc power analysis demonstrated that the sample size was sufficient for detecting significant differences, future studies with pre-defined sample size calculations are warranted to further validate our findings. The seventh limitation is the absence of follow-up beyond 30 days. Readmissions and late mortality could not be captured in this study, which may have impacted the generalizability of our findings regarding long-term outcomes.

## CONCLUSIONS

This study highlights the association between low MAP duration and mortality in CIPs. When the duration of MAP remaining below 65 mmHg exceeds 135 minutes, the risk of 30-day mortality significantly increases. Additionally, if this duration surpasses three hours, the risk of 48-hour mortality rises dramatically. These findings support incorporating the monitoring of cumulative hypotension duration (ΔtMAP<65) into routine emergency department protocols for critically ill patients, as it may guide earlier interventions and improve patient survival. Further research is needed to validate these thresholds in larger populations and to examine if targeted interventions to minimize ΔtMAP<65 can lower mortality in CIPs.

### Authors’ Contributions:

**MCD:** Conceptualization, Methodology, Investigation, Writing - review & editing, Writing - original draft, Supervision, Project administration.

**ES:** Conceptualization, Methodology, Software, Data curation, Formal analysis, Visualization, Writing - review & editing, Writing - original draft, Supervision. All authors read and approved the final version.

**KS:** Writing - review & editing, Writing - original draft, Methodology, Data curation, Formal analysis, Investigation.

All authors have read and approved the final version and they are also responsible and accountable for the accuracy and integrity of the work.
